# Modeling the effects of contact tracing on COVID-19 transmission

**DOI:** 10.1186/s13662-020-02972-8

**Published:** 2020-09-21

**Authors:** Ali Traoré, Fourtoua Victorien Konané

**Affiliations:** Laboratoire de Mathématiques et Informatique (LAMI), Département de Mathématiques, Université Joseph KI-ZERBO, 03 BP 7021 Ouagadougou 03 Ouagadougou, Burkina Faso

**Keywords:** 34D20, 34D23, 34D45, 37C75, COVID-19, Mathematical model, Stability, Lyapunov function, Contact tracing

## Abstract

In this paper, a mathematical model for COVID-19 that involves contact tracing is studied. The *contact tracing-induced* reproduction number $\mathcal{R}_{q}$ and equilibrium for the model are determined and stabilities are examined. The global stabilities results are achieved by constructing Lyapunov functions. The *contact tracing-induced* reproduction number $\mathcal{R}_{q}$ is compared with the basic reproduction number $\mathcal{R}_{0}$ for the model in the absence of any intervention to assess the possible benefits of the contact tracing strategy.

## Introduction

The coronavirus belongs to a large family of viruses that can cause several diseases for humans such as common cold and SARS. Recently, in December 2019, the World Health Organization was alerted to several causes of pneumonia in Wuhan, China. Furthermore, it has been proved that this new infection is caused by a new virus from the coronavirus family called COVID-19. From then on, this disease has become a serious health problem. Confirmed cases have been reported in more than 210 countries. This disease is transmitted by having a contact with an infected person through droplets when a person coughs or sneezes. Symptoms of COVID-19 are similar to those of SARS-Cov and MERS-Cov and typically dry cough, fever, fatigue, breathing difficulty [1]. Due to the lack of specific anti-COVID-19 therapeutic treatment and effective vaccine, some interventions have been implemented by many countries to prevent and control the disease. Among these interventions, we have travel restrictions and massive quarantine. Contact tracing is also one of the implemented measure that consists to keep track of people who had direct contact with the COVID-19 patients. Contacts are monitored for signs of illness within 14 days. In the case of apparition of the symptoms related to the disease, they are isolated, tested and hospitalized. Several mathematical models related to the COVID-19 epidemic have been studied (see [2–18]). In [18], Imai et al. conducted computational modeling to establish the size of the disease outbreak in Wuhan. They show that control measures need to block well over 60% of transmission to be effective in containing disease outbreak. Tang et al. [5] established that intensive contact tracing followed by quarantine and isolation can effectively reduce the transmission risk of COVID-19. Thus, it becomes important for health authorities to know the contact tracing coverage rate that could be required for the disease control or elimination.

In the present paper, we propose a complete mathematical analysis of a COVID-19 model that includes contact tracing measures. A stability analysis is performed to study the epidemiological consequences of this control strategy. Specifically, we propose to determine the threshold parameter that measure initial disease transmission and to analyze the steady state stability. This basic reproduction number is used to compute the potential impact of contact tracing on the spread of COVID-19.

## Model and preliminary results

We proposed a model that describes the transmission dynamics of COVID-19. Based on the information about the disease progression, we subdivided the human population into five compartments, namely, the susceptible population (*S*), the infectious without symptoms or exposed population (*E*), the quarantined exposed population ($E_{q}$), the infectious with symptoms or infected population (*I*) and the recovered population (*R*). New individual enter into susceptible population according to rate Λ. A susceptible human has a contact with an exposed or an infected human at rate *k*. When this contact happens, the probability that this susceptible gets infection from an exposed or infected human is $p_{es}$ and $p_{is}$, respectively. Furthermore, we assume that contact tracing is implemented by the health authorities. Beyond these investigations a proportion of *q* of individuals exposed to the virus is quarantined and the remain proportion $1-q$ is missed from the contact tracing. *μ* design the human natural death rate, *α* is the disease-induced death rate, $\sigma ^{-1}$ is the incubation period between the infection and the onset of symptoms and *γ* is the rate of recovery from infection. The dynamics of the different populations is given by the following system of differential equations:

1 $$ \textstyle\begin{cases} \frac{dS}{dt}=\Lambda -kp_{es} SE-kp_{is} SI-\mu S, \\ \frac{dE}{dt}=(1-q)kp_{es}SE+(1-q)kp_{is}SI-(\mu + \sigma )E, \\ \frac{dI}{dt}=\sigma E-(\mu +\gamma +\alpha )I, \\ \frac{dE_{q}}{dt}=qkp_{es}SE+qkp_{is}SI-\mu E_{q}, \\ \frac{dR}{dt}= \gamma I -\mu R. \end{cases} $$

Since the recovered human population *R* does not appear in the remaining equations of system (1), it is then sufficient to consider the following system:

2 $$ \textstyle\begin{cases} \frac{dS}{dt}=\Lambda -kp_{es} SE-kp_{is} SI-\mu S, \\ \frac{dE}{dt}=(1-q)kp_{es}SE+(1-q)kp_{is}SI-(\mu + \sigma )E, \\ \frac{dI}{dt}=\sigma E-(\mu +\gamma +\alpha )I, \\ \frac{dE_{q}}{dt}=qkp_{es}SE+qkp_{is}SI-\mu E_{q}, \end{cases} $$

with initial conditions

$$\begin{aligned} \bigl(S(0),E(0),I(0),E_{q}(0) \bigr) \in \mathbf{R}^{4}_{+}. \end{aligned}$$

By considering the system on the faces of $\mathbf{R}^{4}_{+}$, we obtain

$$\begin{aligned} \textstyle\begin{cases} \frac{dS}{dt} | _{S=0}=\Lambda \geq 0, \\ \frac{dE}{dt} | _{E=0}=(1-q)kp_{is}SI\geq 0, \\ \frac{dI}{dt} | _{I=0}=\sigma E\geq 0, \\ \frac{dE_{q}}{dt} | _{E_{q}=0}=qkp_{is}SI\geq 0. \end{cases}\displaystyle \end{aligned}$$

Thus, Proposition 2.1 of [19] implies that each solution of (2) remains in $\mathbf{R}^{4}_{+}$.

Further, we denote by $N=S+E+I+E_{q}$ the human total population. Thus, it follows that

$$\begin{aligned} \frac{dN}{dt}\leq \Lambda -\mu N, \end{aligned}$$

that is,

3 $$ \limsup_{t \rightarrow \infty }N \leq \frac{ \Lambda }{\mu }, $$

which shows that all solutions of (2) are bounded.

Thus, the suitable region for system (2) is

$$\begin{aligned} \mathcal{D}= \biggl\{ (S,E,I,E_{q}) \in \mathbf{R}_{+}^{4} : N \leq \frac{ \Lambda }{\mu } \biggr\} . \end{aligned}$$

It can be verified that the compact region $\mathcal{D}$ is positively invariant and attracting under the flow described by (2).

For the rest of the paper, we will study the dynamics of model (2) in $\mathcal{D}$.

### Proposition 2.1

*For every initial value in* $\mathbf{R}_{+}^{4}$, *solutions of system* (2) *exist for all time* $t>0$.

### Proof

Since the right-hand side of system (2) is locally Lipschitz, the local existence follows. Since $\mathcal{D}$ is positively invariant and attracting, all solutions of (2) are bounded, which gives us the global existence. This completes the proof. □

## Stability of the disease-free equilibrium

By direct calculation we show that model (2) has a disease-free equilibrium given by

$$\begin{aligned} \mathcal{E}_{0}=\bigl(S^{0},E^{0},I^{0},E_{q}^{0} \bigr) = \biggl( \frac{\Lambda }{\mu },0,0,0 \biggr). \end{aligned}$$

The new infection matrix *F* and the transition matrix *V* are given by

$$\begin{aligned} F= \begin{pmatrix} (1-q)kp_{es}\frac{\Lambda }{\mu }& (1-q)kp_{is}\frac{\Lambda }{\mu } \\ 0&0 \end{pmatrix}, \qquad V= \begin{pmatrix} \mu +\sigma &0 \\ -\sigma &\mu +\gamma + \alpha \end{pmatrix}. \end{aligned}$$

The *contact tracing-induced* reproduction number $\mathcal{R}_{q}$ of model (2) is then defined as the spectral radius of the next generation matrix $FV^{-1}$ (see [20]), that is,

$$\begin{aligned} \mathcal{R}_{q}= \rho \bigl(FV^{-1}\bigr)= \frac{k\Lambda (1-q)}{\mu (\sigma +\mu )} \biggl(p_{es}+ \frac{\sigma p_{is}}{\mu +\gamma +\alpha } \biggr). \end{aligned}$$

The local stability result is stated as follows.

### Theorem 3.1

*If* $\mathcal{R}_{q} \leq 1$, *the disease*-*free equilibrium* $\mathcal{E}_{0}$*is locally asymptotically stable*.

### Proof

Let $\mathcal{R}_{q} \leq 1$. It is sufficient to prove that the eigenvalues of Jacobian matrix of system (2) evaluated at $\mathcal{E}_{0}$ have negative real parts.

Direct calculation shows that these eigenvalues are solutions of the equation

$$\begin{aligned} (\lambda +\mu )^{2}(\lambda +\alpha +\gamma +\mu )\mathcal{Q}( \lambda )=0, \end{aligned}$$

where

$$\begin{aligned} \mathcal{Q}(\lambda )= \biggl(\lambda +\mu +\sigma - \frac{ (1-q)kp_{es}\Lambda }{\mu }\biggr) ( \lambda +\gamma +\alpha +\mu )- \frac{\sigma (1-q)kp_{is}\Lambda }{\mu }. \end{aligned}$$

Clearly the proof will be achieved if the roots of $\mathcal{Q}(\lambda )$ have negative real parts. By contradiction, let assume that at least one root of $\mathcal{Q}(\lambda )$ denoted by $\lambda _{0}$ has a positive real part. Then

$$\begin{aligned} \biggl(\lambda _{0}+\mu +\sigma - \frac{ (1-q)kp_{es}\Lambda }{\mu }\biggr) (\lambda _{0}+\gamma +\alpha +\mu )= \frac{\sigma (1-q)kp_{IS}\Lambda }{\mu }. \end{aligned}$$

By applying the absolute value it follows that

$$\begin{aligned} \biggl\vert \biggl(\lambda _{0}+\mu +\sigma - \frac{ (1-q)kp_{es}\Lambda }{\mu } \biggr) (\lambda _{0}+\gamma +\alpha +\mu ) \biggr\vert =& \frac{\sigma (1-q)kp_{is}\Lambda }{\mu } \\ >&(\gamma +\alpha +\mu ) \\ &{}\times \biggl\vert \mu +\sigma - \frac{ (1-q)kp_{es}\Lambda }{\mu } \biggr\vert . \end{aligned}$$

Thus,

$$\begin{aligned} \frac{\sigma (1-q)kp_{is}\Lambda }{\mu (\mu +\gamma +\alpha )}> \biggl\vert \mu +\sigma - \frac{ (1-q)kp_{es}\Lambda }{\mu } \biggr\vert >\mu + \sigma - \frac{ (1-q)kp_{es}\Lambda }{\mu }. \end{aligned}$$

This yields

$$\begin{aligned} \frac{\sigma (1-q)kp_{is}\Lambda }{\mu (\mu +\gamma +\alpha )}+ \frac{ (1-q)kp_{es}\Lambda }{\mu }>\mu +\sigma . \end{aligned}$$

So, we have

$$\begin{aligned} \mathcal{R}_{q}= \frac{\sigma (1-q)kp_{is}\Lambda }{\mu (\mu +\sigma )(\gamma +\alpha +\mu )} + \frac{ (1-q)kp_{es}\Lambda }{\mu (\mu +\sigma )}>1, \end{aligned}$$

which is a contradiction. Hence, $\mathcal{E}_{0}$ is locally asymptotically stable. This completes the proof. □

What follows now states the global behavior result of disease-free equilibrium.

### Theorem 3.2

*The disease*-*free equilibrium* $\mathcal{E}_{0}$*is globally asymptotically stable in* $\mathcal{D}$*if* $\mathcal{R}_{q}<1$*and unstable if* $\mathcal{R}_{q}>1$.

### Proof

Set $X=(E,I)^{T}$ and $u= ((1-q)kp_{es}, (1-q)kp_{is} )$.

We can verify that

$$\begin{aligned} \frac{dX}{dt} \leq (F-V)X, \end{aligned}$$

where *F* and *V* are given at the beginning of this section.

By doing some algebraic computation, it then follows that

$$\begin{aligned} \rho \bigl(V^{-1}F\bigr)=\rho \bigl(FV^{-1}\bigr)= \mathcal{R}_{q} \end{aligned}$$

and

$$\begin{aligned} uV^{-1}F= \mathcal{R}_{q} u. \end{aligned}$$

Thus, *u* is a left eigenvector associated with the eigenvalue $\mathcal{R}_{q}$ of the matrix $V^{-1}F$.

We now consider the following Lyapunov function

$$\begin{aligned} \mathcal{L}=uV^{-1}X. \end{aligned}$$

Taking the differentiation of $\mathcal{L}$ with respect to *t*, we obtain

$$\begin{aligned} \frac{d\mathcal{L}}{dt}=uV^{-1} \frac{dX}{dt}. \end{aligned}$$

Thus,

$$\begin{aligned} \frac{d\mathcal{L}}{dt}\leq uV^{-1}(F-V)X=u(\mathcal{R}_{q}-1)X. \end{aligned}$$

If $\mathcal{R}_{q}<1$, the equality $\frac{d\mathcal{L}}{dt}= 0$ implies that $uX=0$. This leads to $E=I=0$ by noting that all components of *u* are positive. Hence, when $\mathcal{R}_{q}<1$, setting the right-hand side of (2) equal to zero and replacing *E* and *I* by 0 yields $S=S_{0}=\frac{\Lambda }{\mu }$, and $E_{q}=E=I=0$. The invariant set on which $\frac{d\mathcal{L}}{dt}= 0$ contains only the singleton $\{ \mathcal{E}_{0} \}$. Therefore, by LaSalle’s invariant principle (see [21]), $\mathcal{E}_{0}$ is globally asymptotically stable in $\mathcal{D}$.

If $\mathcal{R}_{q}>1$, then it follows from the continuity of the vector fields that $\frac{d\mathcal{L}}{dt}>0$ in a neighborhood of the interior of $\mathcal{D}$. Thus, $\mathcal{E}_{0}$ is unstable by the Lyapunov stability theory. This completes the proof. □

## Global stability of the endemic equilibrium

Direct calculation shows that model (2) have a positive endemic equilibrium $\mathcal{E}_{*}=(S^{*},E^{*},I^{*},E_{q}^{*})$ when $\mathcal{R}_{q} > 1$, where

4 $$ \textstyle\begin{cases} S^{*}= \frac{(\sigma +\mu )(\mu +\gamma +\alpha )}{k(1-q)( p_{es}(\mu +\gamma +\alpha )+p_{is}\sigma )}, \\ E^{*}= \frac{ \mu ^{2}(\mu +\gamma +\alpha )(\mathcal{R}_{q} -1)}{kp_{is} \sigma + k\mu p_{es}(\mu +\gamma +\alpha ) }, \\ I^{*}= \frac{\sigma \mu ^{2}(\mathcal{R}_{q} -1)}{kp_{is} \sigma + k\mu p_{es}(\mu +\gamma +\alpha ) }, \\ E_{q}^{*} = \frac{q(\sigma +\mu ) }{ (1-q)}\times \frac{ \mu (\mu +\gamma +\alpha )(\mathcal{R}_{q} -1)}{kp_{is} \sigma + k\mu p_{es}(\mu +\gamma +\alpha ) }. \end{cases} $$

### Theorem 4.1

*If* $\mathcal{R}_{q} > 1$, *the endemic equilibrium* $\mathcal{E}_{*}$*is globally asymptotically stable*.

### Proof

Setting the right-hand side of (2) equal to 0 at $\mathcal{E}_{*}$ yields

5 $$\begin{aligned}& \Lambda =kp_{es} S^{*}E^{*}+kp_{is} S^{*}I^{*}+\mu S^{*}, \end{aligned}$$

6 $$\begin{aligned}& \mu +\sigma =(1-q) \frac{kp_{es} S^{*}E^{*}+kp_{is} S^{*}I^{*}}{E^{*}}, \end{aligned}$$

7 $$\begin{aligned}& \mu = \frac{qkp_{es}S^{*}E^{*}+qkp_{is}S^{*}I^{*}}{ E_{q}^{*}}, \end{aligned}$$

8 $$\begin{aligned}& \mu +\gamma +\alpha = \frac{\sigma E^{*}}{I^{*}}. \end{aligned}$$

Let *f* be a function defined by $f : \mathbf{R}_{+}^{*} \rightarrow \mathbf{R}_{+}$, $f(x)=x-1-\ln x$.

The function *f* has a strict global minimum $f(1)=0$.

Furthermore, we set $\mathcal{G}(y)=\int _{y^{*}}^{y}\frac{x-y^{*}}{x}\,dx$ for $y>0$, where $y^{*}>0$, and *y* can be replaced by *S*, *E*, *I* or $E_{q}$.

We define the Lyapunov function $\mathcal{V}$ by

$$\begin{aligned} \mathcal{V}=c_{1} \mathcal{G}(S)+c_{2} \mathcal{G}(E)+c_{3} \mathcal{G}(E_{q})+c_{4} \mathcal{G}(I), \end{aligned}$$

where

$$\begin{aligned} c_{1}=\frac{1}{kp_{es} S^{*}E^{*} +kp_{is} S^{*}I^{*}}, \quad\quad c_{2}= \frac{1}{(1-q)c_{1}}, \qquad c_{3}=\frac{1}{q c_{1}},\qquad c_{4}= \frac{kp_{is}S^{*}I^{*} }{\sigma E^{*}}. \end{aligned}$$

Clearly $\mathcal{V}$ is non-negative and strictly minimized at the endemic equilibrium $\mathcal{E}_{*}$. To avoid long expression, the derivatives of $\mathcal{G}(S)$, $\mathcal{G}(E)$, $\mathcal{G}(E_{q})$ and $\mathcal{G}(I)$ with respect to *t* will be calculated separately and combined to get that of $\mathcal{V}$.

We compute $\frac{d \mathcal{G}(S)}{dt}$ as follows:

9 $$\begin{aligned} \frac{d \mathcal{G}(\mathcal{S})}{dt} =& \biggl(1-\frac{S^{*}}{S} \biggr) \frac{dS}{dt} \\ =& \biggl(1-\frac{S^{*}}{S} \biggr) ( \Lambda -kp_{es} SE-kp_{is} SI- \mu S ). \end{aligned}$$

We replace Λ in (9) by using Eq. (5) to obtain

10 $$\begin{aligned} \frac{d \mathcal{G}(\mathcal{S})}{dt} =& \biggl(1-\frac{S^{*}}{S} \biggr) \bigl( kp_{es} S^{*}E^{*}+kp_{is} S^{*}I^{*}+\mu S^{*}-kp_{es} SE-kp_{is} SI-\mu S \bigr) \\ =& \biggl(1-\frac{S^{*}}{S} \biggr) \biggl( kp_{es} S^{*}E^{*}\biggl( 1- \frac{S}{S^{*}} \frac{E}{E^{*}} \biggr) \\ & {} + kp_{is} S^{*}I^{*}\biggl(1- \frac{S}{S^{*}} \frac{I}{I^{*}} \biggr)+\mu S^{*}\biggl(1- \frac{S}{S^{*}}\biggr) \biggr) \\ =& -\mu \frac{ (S-S^{*})^{2}}{S} +kp_{es} S^{*}E^{*} \biggl( 1 - \frac{S^{*}}{S}- \frac{S}{S^{*}} \frac{E}{E^{*}} + \frac{E}{E^{*}} \biggr) \\ & {} + kp_{is} S^{*}I^{*} \biggl( 1 - \frac{S^{*}}{S}- \frac{S}{S^{*}} \frac{I}{I^{*}} + \frac{I}{I^{*}} \biggr) \\ =&-\mu \frac{ (S-S^{*})^{2}}{S}+ kp_{es} S^{*}E^{*} \biggl( f \biggl( \frac{E}{E^{*}} \biggr)- f \biggl( \frac{S^{*}}{S} \biggr) - f \biggl( \frac{S}{S^{*}} \frac{E}{E^{*}} \biggr) \biggr) \\ & {} + kp_{is} S^{*}I^{*} \biggl(f \biggl( \frac{I}{I^{*}} \biggr)- f \biggl( \frac{S^{*}}{S} \biggr) - f \biggl( \frac{S}{S^{*}} \frac{I}{I^{*}} \biggr) \biggr). \end{aligned}$$

The derivative $\frac{d \mathcal{G}(E)}{dt}$ is given by

11 $$\begin{aligned} \frac{d \mathcal{G}(E)}{dt} =& \biggl(1-\frac{E^{*}}{E} \biggr) \frac{dE}{dt} \\ =& \biggl(1-\frac{E^{*}}{E} \biggr) \bigl( (1-q)kp_{es} SE+(1-q)kp_{is} SI-( \mu +\sigma )E \bigr). \end{aligned}$$

Replacing $\mu +\sigma $ in (11) by using (6), we get

12 $$\begin{aligned} \frac{d \mathcal{G}(E)}{dt} =& \biggl(1-\frac{E^{*}}{E} \biggr) \biggl( (1-q)kp_{es} SE+(1-q)kp_{is} SI \\ & {} - (1-q)k\bigl(p_{es} S^{*}E^{*}+p_{is} S^{*}I^{*}\bigr) \frac{E}{E^{*}} \biggr) \\ =& \biggl(1-\frac{E^{*}}{E} \biggr) \biggl( (1-q)kp_{es} S^{*}E^{*}\biggl( \frac{S}{S^{*}} \frac{E}{E^{*}}- \frac{E}{E^{*}} \biggr) \\ & {} + (1-q)kp_{is} S^{*}I^{*}\biggl( \frac{S}{S^{*}} \frac{I}{I^{*}} - \frac{E}{E^{*}} \biggr) \biggr) \\ =&(1-q)kp_{es} S^{*}E^{*} \biggl( \frac{S}{S^{*}} \frac{E}{E^{*}} - \frac{E}{E^{*}} - \frac{S}{S^{*}}+1 \biggr) \\ & {} + (1-q)kp_{is} \biggl( \frac{S}{S^{*}} \frac{I}{I^{*}} - \frac{E}{E^{*}} - \frac{E^{*}}{E} \frac{S}{S^{*}} \frac{I}{I^{*}}+1 \biggr) \\ =&(1-q)kp_{es} S^{*}E^{*} \biggl( f \biggl( \frac{S}{S^{*}} \frac{E}{E^{*}} \biggr)- f \biggl( \frac{E}{E^{*}} \biggr) - f \biggl( \frac{S}{S^{*}} \biggr) \biggr) \\ & {} + (1-q)kp_{is} S^{*}I^{*} \biggl( f \biggl( \frac{S}{S^{*}} \frac{I}{I^{*}} \biggr)- f \biggl( \frac{E}{E^{*}} \biggr) - f \biggl( \frac{E^{*}}{E} \frac{S}{S^{*}} \frac{I}{I^{*}} \biggr) \biggr). \end{aligned}$$

$\frac{d \mathcal{G}(E_{q})}{dt}$ is computed as follows:

13 $$\begin{aligned} \frac{d \mathcal{G}(E_{q})}{dt} =& \biggl(1-\frac{E_{q}^{*}}{E_{q}} \biggr) \frac{dE_{q}}{dt} \\ =& \biggl(1-\frac{E_{q}^{*}}{E_{q}} \biggr) ( qkp_{es} SE+qkp_{is} SI- \mu E_{q} ). \end{aligned}$$

In (13), *μ* is replaced by (7) to obtain

14 $$\begin{aligned} \frac{d \mathcal{G}(E_{q})}{dt} =& \biggl(1-\frac{E_{q}^{*}}{E_{q}} \biggr) \biggl( qkp_{es} SE+qkp_{is} SI- \frac{qkp_{es}S^{*}E^{*}+qkp_{is}S^{*}I^{*}}{ E_{q}^{*}} E_{q} \biggr) \\ =& \biggl(1-\frac{E_{q}^{*}}{E_{q}} \biggr) \biggl( qkp_{es}S^{*}E^{*} \biggl( \frac{S}{S^{*}}\frac{E}{E^{*}}-\frac{E_{q}}{E_{q}^{*}} \biggr)+qkp_{is}S^{*}I^{*} \biggl( \frac{S}{S^{*}}\frac{I}{I^{*}}-\frac{E_{q}}{E_{q}^{*}} \biggr) \biggr) \\ =&qkp_{es}S^{*}E^{*} \biggl( \frac{S}{S^{*}} \frac{E}{E^{*}}- \frac{E_{q}}{E_{q}^{*}}- \frac{E_{q}^{*}}{E_{q}}\frac{S}{S^{*}} \frac{E}{E^{*}}+1 \biggr)+qkp_{is}S^{*}I^{*} \biggl( \frac{S}{S^{*}} \frac{I}{I^{*}} \\ & {} - \frac{E_{q}}{E_{q}^{*}}- \frac{E_{q}^{*}}{E_{q}}\frac{S}{S^{*}} \frac{I}{I^{*}}+1 \biggr) \\ =&qkp_{es}S^{*}E^{*} \biggl( f \biggl( \frac{S}{S^{*}}\frac{E}{E^{*}} \biggr)-f \biggl(\frac{E_{q}}{E_{q}^{*}} \biggr)- f \biggl(\frac{E_{q}^{*}}{E_{q}} \frac{S}{S^{*}}\frac{E}{E^{*}} \biggr) \biggr) \\ & {} + qkp_{is}S^{*}I^{*} \biggl( f \biggl( \frac{S}{S^{*}}\frac{I}{I^{*}} \biggr) -f \biggl(\frac{E_{q}}{E_{q}^{*}} \biggr)- \biggl( \frac{E_{q}^{*}}{E_{q}}\frac{S}{S^{*}}\frac{I}{I^{*}} \biggr) \biggr). \end{aligned}$$

$\frac{d \mathcal{G}(I)}{dt}$ is given by

15 $$\begin{aligned} \frac{d \mathcal{G}(I)}{dt} =& \biggl(1-\frac{I^{*}}{I} \biggr) \frac{dI}{dt} \\ =& \biggl(1-\frac{I^{*}}{I} \biggr) \bigl( \sigma E -(\mu +\gamma +\alpha )I \bigr). \end{aligned}$$

In (15), $\mu +\gamma +\alpha $ is replaced by (8) to get

16 $$\begin{aligned} \frac{d \mathcal{G}(I)}{dt} =& \biggl(1-\frac{I^{*}}{I} \biggr) \biggl( \sigma E -\sigma E^{*} \frac{I}{I^{*}} \biggr) \\ =&\sigma E^{*} \biggl(1-\frac{I^{*}}{I} \biggr) \biggl( \frac{E}{E^{*}}- \frac{I}{I^{*}} \biggr) \\ =&\sigma E^{*} \biggl(\frac{E}{E^{*}}- \frac{I}{I^{*}}- \frac{I^{*}}{I}\frac{E}{E^{*}} \biggr) \\ =&\sigma E^{*} \biggl( f \biggl(\frac{E}{E^{*}} \biggr)- f \biggl( \frac{I}{I^{*}} \biggr)- f \biggl( \frac{I^{*}}{I} \frac{E}{E^{*}} \biggr) \biggr). \end{aligned}$$

By multiplying Eqs. (10), (12), (14) and (16) by the coefficients $c_{1}$, $c_{2}$, $c_{3}$ and $c_{4}$, respectively, and after a rearrangement we get

$$\begin{aligned} \frac{d\mathcal{V}}{dt} =&- \frac{\mu (S-S^{*})^{2}}{ S(kp_{es} S^{*}E^{*} +kp_{is} S^{*}I^{*})} - \frac{p_{es} E^{*}}{p_{es} E^{*} +p_{is}I^{*}} f \biggl( \frac{S}{S^{*}} \frac{E}{E^{*}} \biggr) \\ & {} - \frac{p_{is} I^{*}}{p_{es} E^{*} +p_{is} I^{*}}f \biggl( \frac{S}{S^{*}} \frac{I}{I^{*}} \biggr) - \frac{p_{es} E^{*}}{ p_{es} E^{*} +p_{is} I^{*}} f \biggl( \frac{S}{S^{*}} \biggr) \\ & {} - \frac{p_{is} I^{*}}{ p_{es} E^{*} +p_{is} I^{*}} f \biggl( \frac{E^{*}}{E} \frac{S}{S^{*}} \frac{I}{I^{*}} \biggr) -kp_{is} S^{*}I^{*} \biggl( f \biggl( \frac{I}{I^{*}} \biggr)+ f \biggl( \frac{I^{*}}{I} \frac{E}{E^{*}} \biggr) \biggr) \\ & {} - \frac{p_{es}E^{*}}{p_{es} E^{*} +p_{is} I^{*}} f \biggl( \frac{E_{q}^{*}}{E_{q}}\frac{S}{S^{*}} \frac{E}{E^{*}} \biggr)- \frac{p_{is}I^{*}}{p_{es} E^{*} +p_{is} I^{*}} \biggl( \frac{E_{q}^{*}}{E_{q}} \frac{S}{S^{*}}\frac{I}{I^{*}} \biggr) \\ & {} - f \biggl( \frac{S^{*}}{S} \biggr) -f \biggl( \frac{E_{q}}{E_{q}^{*}} \biggr). \end{aligned}$$

That is,

$$\begin{aligned} \frac{d \mathcal{V}}{dt} \leq 0. \end{aligned}$$

The equality $\frac{d\mathcal{V}}{dt}=0$ is fulfilled if and only if $(S,E,I,E_{q})=(S^{*},E^{*},I^{*},E_{q}^{*})$. The largest invariant subset of $\frac{d \mathcal{V}}{dt}=0$ is the singleton $\{ \mathcal{E}_{*}\}$. Thus, by LaSalle’s invariant principle (see [21]), $\mathcal{E}_{*}$ is globally asymptotically stable. This completes the proof. □

## Numerical results

### Graphical representation of the model

**Figure 1:**
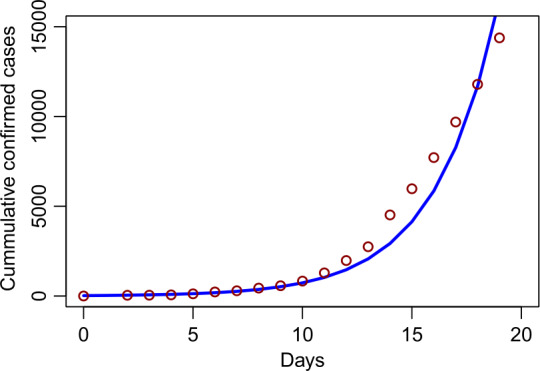
Cumulative confirmed cases for mainland China from January 15, 2020 to February 02, 2020 (see [22] for the data). Red circles denote the reported cases and solid blue line denotes the simulation result. The *contact tracing-induced* reproduction number is $\mathcal{R}_{q}=5.63$ based on the parameters from Table 1

Numerical simulations are carried out to support the analytical results. Figure 1 shows the numbers of cumulative confirmed cases in mainland China from January 15, 2020 to February 02, 2020 (see [22] for the data). The parameters used are presented in Table 1 and the initial condition is given by Table 2. Based on the parameters values, we found that the *contact tracing-induced* reproduction number is $\mathcal{R}_{q}=5.63$.

**Table 1:** Definition and values of model parameters

Parameters	Definition	Value	Source
Λ	Recruitment rate	271.23 day^−1^	[23]
*k*	Contact rate	10.582 day^−1^	[24]
$p_{es}$	Transmission probability from *E* to *S*	1.5 × 10^−14^ day^−1^	[24]
$p_{is}$	Transmission probability from *I* to *S*	2.010 × 10^−8^ day^−1^	[24]
*μ*	Natural death rate	3.01 × 10^−5^ day^−1^	[23]
*q*	Quarantine proportion of exposed individuals *E*	10^−5^ day^−1^	[24]
1/*σ*	Incubation period	7 days	[25]
*γ*	Removal rate	0.33 day^−1^	[5]
*α*	Disease-induced death rate	0.01 day^−1^	[23]

**Table 2:** Definition and initial values of variables of the model

Parameters	Definition	Initial value	Source
*S*	Susceptible population	11,081,000	[5, 26]
*E*	Exposed population	118.552	[24]
*I*	Infected population with symptomatic	20	[24]
$E_{q}$	Quarantined exposed population	1	[24]

**Figure 2:**
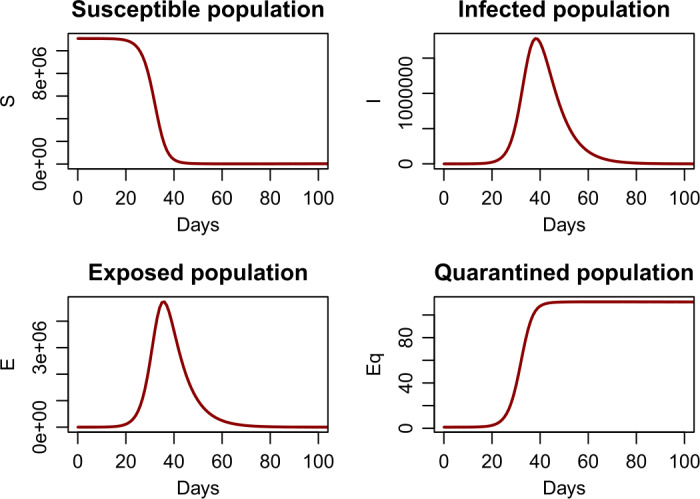
A simulation result for the outbreak size in mainland China using the parameters from Table 1 and initial values from Table 2

Figure 2 is a graphical representation that provide a prediction for *S* (the susceptible individuals), *E* (the exposed individuals), *I* (the infected individuals) and $E_{q}$ (the quarantined exposed individuals) using model (2). Results shows that the infection will reach a peak value for about 40 days and then go down afterwards. The long-term behavior of the epidemic is determined by the property of the endemic equilibrium of the system, which is found to be $(S^{*},E^{*},I^{*},E_{q}^{*})=(1{,}599{,}001,1561,656,74)$.

### Effects of contact tracing on the disease control

In this section we explore the effect of contact tracing in the disease transmission. Without contact tracing measure, the model (2) is reduced to the following system of equations:

17 $$ \textstyle\begin{cases} \frac{dS}{dt}=\Lambda -kp_{es} SE-kp_{is} SI-\mu S, \\ \frac{dE}{dt}=kp_{es}SE+ kp_{is}SI-(\mu +\sigma )E, \\ \frac{dI}{dt}=\sigma E-(\mu +\gamma +\alpha )I. \end{cases} $$

The model (17) has a disease-free equilibrium $(\Lambda /\mu ,0,0)$. The new infection matrix $F_{1}$ and the transition matrix $V_{1}$ are given by

$$\begin{aligned} F_{1}= \begin{pmatrix} kp_{es}\frac{\Lambda }{\mu }& kp_{is}\frac{\Lambda }{\mu } \\ 0&0 \end{pmatrix}, \qquad V_{1}= \begin{pmatrix} \mu +\sigma &0 \\ -\sigma &\mu +\gamma + \alpha \end{pmatrix}. \end{aligned}$$

The basic reproduction number $\mathcal{R}_{0}$ of model (17) is then defined as the spectral radius of the next generation matrix $F_{1}V_{1}^{-1}$ (see [20]), that is,

$$\begin{aligned} \mathcal{R}_{0}= \frac{k\Lambda }{\mu (\sigma +\mu )} \biggl(p_{es}+ \frac{\sigma p_{is}}{\mu +\gamma +\alpha } \biggr). \end{aligned}$$

The basic reproduction number $\mathcal{R}_{0}$ measures the power of a disease to invade a population under conditions that facilitate maximal growth.

The *contact tracing-induced* reproduction number $\mathcal{R}_{q}$ can be rewritten as

$$\begin{aligned} \mathcal{R}_{q}=(1-q)\mathcal{R}_{0}. \end{aligned}$$

Note that $1-q$ is the factor by which the contact tracing strategy reduces the number of secondary infections if adopted in a community.

If $\mathcal{R}_{0}<1$, COVID-19 cannot develop into an endemic, and contact tracing strategy may not be necessary. For $\mathcal{R}_{0}>1$, we want to know the necessary condition for slowing the development of COVID-19. Following Hsu Schmitz [27] we have

$$\begin{aligned} \Delta _{E}=\mathcal{R}_{0}-\mathcal{R}_{q}=q \mathcal{R}_{0}. \end{aligned}$$

Clearly, if $q>0$ then $\Delta _{E}>0$.

Differentiating $\mathcal{R}_{q}$ with respect to *q* yields

$$\begin{aligned} \frac{\partial \mathcal{R}_{q} }{\partial q}=-\mathcal{R}_{0}< 0. \end{aligned}$$

The conditions $\Delta _{E}>0$, $\frac{\partial \mathcal{R}_{q} }{\partial q}< 0 $ for slowing down the epidemic are satisfied for $0< q<1$.

Setting the *contact tracing-induced* reproduction number $\mathcal{R}_{q}=1$ and solving for *q* gives the threshold proportion of contact tracing as follows:

$$\begin{aligned} q_{c}=1- 1/\mathcal{R}_{0}. \end{aligned}$$

Contact tracing strategy would succeed in controlling the epidemic ($\mathcal{R}_{q} <1$) if $q>q_{c}$. We observe that $q_{c}$ is increasing function of $\mathcal{R}_{0}$, thus for a population where $\mathcal{R}_{0}$ is large, a high value of $q_{c}$ is required to control COVID-19 using the contact tracing strategy. We conclude that in a population where $\mathcal{R}_{0}$ is large, COVID-19 may not be controlled using the contact tracing measure alone because the corresponding value of $q_{c}$ required is high and perhaps unattainable for such populations.

## Summary and conclusion

We formulated and analyzed a mathematical model for COVID-19 epidemic with contact tracing. Five sub populations were considered: susceptible, exposed, quarantine exposed, infected with symptoms and recovered. Mathematical properties of the model are analyzed.

We computed the *contact tracing-induced* reproduction number $\mathcal{R}_{q}$ of the model. We proved that when $\mathcal{R}_{q}< 1$, the disease-free state $\mathcal{E}_{0}$ is globally asymptotically stable. We also established that when $\mathcal{R}_{q}> 1$, the unique endemic equilibrium $\mathcal{E}_{*}$ is globally asymptotically stable.

The analysis of the model illustrates that the contact tracing strategy can reduce the basic reproduction number $\mathcal{R}_{0}$ (without contact tracing strategy) to values below unity as intended for disease control, thus one would succeed in controlling the epidemic. The results obtained in this paper show that effective control of the epidemic can be achieved when the effectiveness of contact tracing is high, and if $\mathcal{R}_{0}$ is not large. Otherwise in the population where $\mathcal{R}_{0}$ is large, the epidemic may not be controlled using a contact tracing strategy alone because the corresponding value $q_{c}$ required is high and perhaps unattainable for such populations. Another interesting feature to study would be to see the effects of various preventive measures. In principle this can be done using the current paper by considering preventive measures affecting some parameters.
